# Preferential X Chromosome Inactivation as a Mechanism to Explain Female Preponderance in Myasthenia Gravis

**DOI:** 10.3390/genes13040696

**Published:** 2022-04-15

**Authors:** Vanessa Nicolì, Silvia Maria Tabano, Patrizia Colapietro, Michelangelo Maestri, Roberta Ricciardi, Andrea Stoccoro, Laura Fontana, Melania Guida, Monica Miozzo, Fabio Coppedè, Lucia Migliore

**Affiliations:** 1 Department of Translational Research and of New Surgical and Medical Technologies, Medical School, University of Pisa, 56126 Pisa, Italy; vanessa.nicoli@med.unipi.it (V.N.); andrea.stoccoro@unipi.it (A.S.); fabio.coppede@unipi.it (F.C.); 2 Department of Pathophysiology and Transplantation, Medical Genetics, University of Milan, 20122 Milan, Italy; silvia.tabano@unimi.it (S.M.T.); patrizia.colapietro@unimi.it (P.C.); 3 Laboratory of Medical Genetics, Ca’ Granda Foundation, Policlinico Hospital, 20122 Milan, Italy; 4 Neurology Unit, Department of Clinical and Experimental Medicine, University of Pisa, 56126 Pisa, Italy; maestri74@gmail.com (M.M.); robertaricciardi@live.it (R.R.); melaniaguida@gmail.com (M.G.); 5 Division of Thoracic Surgery, Department of Surgical, Medical and Molecular Pathology, University of Pisa, 56126 Pisa, Italy; 6 Unit of Medical Genetics, ASST Santi Paolo e Carlo, Department of Health Sciences, University of Milan, 20122 Milan, Italy; laura.fontana@unimi.it (L.F.); monica.miozzo@unimi.it (M.M.); 7 Department of Laboratory Medicine, University Hospital of Pisa, 56124 Pisa, Italy

**Keywords:** myasthenia gravis, X chromosome inactivation, skewed XCI, gender bias, gender medicines, autoimmune disease, neuromuscular disease, HUMARA, epigenetics

## Abstract

Myasthenia gravis (MG) is a neuromuscular autoimmune disease characterized by prevalence in young women (3:1). Several mechanisms proposed as explanations for gender bias, including skewed X chromosome inactivation (XCI) and dosage or sex hormones, are often involved in the development of autoimmunity. The skewed XCI pattern can lead to an unbalanced expression of some X-linked genes, as observed in several autoimmune disorders characterized by female predominance. No data are yet available regarding XCI and MG. We hypothesize that the preferential XCI pattern may contribute to the female bias observed in the onset of MG, especially among younger women. XCI analysis was performed on blood samples of 284 women between the ages of 20 and 82. XCI was tested using the Human Androgen Receptor Assay (HUMARA). XCI patterns were classified as random (XCI < 75%) and preferential (XCI ≥ 75%). In 121 informative patients, the frequency of skewed XCI patterns was 47%, significantly higher than in healthy controls (17%; *p* ≤ 0.00001). Interestingly, the phenomenon was observed mainly in younger patients (<45 years; *p* ≤ 0.00001). Furthermore, considering the XCI pattern and the other clinical characteristics of patients, no significant differences were found. In conclusion, we observed preferential XCI in MG female patients, suggesting its potential role in the aetiology of MG, as observed in other autoimmune diseases in women.

## 1. Introduction

Myasthenia gravis (MG) is a neuromuscular autoimmune condition caused by autoantibodies acting against the neuromuscular junction proteins (NMJ) of striated skeletal muscles. It shows a worldwide prevalence of 150–250 cases per million and an annual incidence of 8–10 cases per million. Like most autoimmune diseases, MG shows a bimodal distribution between the sexes [1,2].

Skeletal muscle weakness and early fatigue are the characteristic hallmarks of the disease. They typically worsen with repeated muscle work and improve after rest [3]. About 60% of patients have ocular symptoms, which manifest as ptosis (hanging of the eyelid) or diplopia (double vision). In 20% of cases, the disease remains limited to the initial extraocular muscle weakness; 80% of patients degenerate to more generalized forms of MG affecting respiratory muscles, arms, or limbs in varying degrees of severity, typically within two years from disease onset [4].

MG is considered a classic example of an antibody-mediated autoimmune disease. Key molecules targeting NMJ by autoantibodies mainly include the acetylcholine receptor (AChR), muscle-specific kinase (MUSK), lipoprotein-related protein 4 (LRP4), and arginine. These molecules are generally used to classify different subsets of the disease [5,6].

Autoantibodies acting against the AChR are found in approximately 80% of patients with MG. Part of the complexity of MG is attributable to the heterogeneity of the AChR+ MG subgroup, which is further subdivided according to genetic, thymic histopathology (thymoma-associated MG: TAMG, the non-thymomatous subtypes, and disease onset (early onset MG: EOMG, late onset MG: LOMG) [7,8]. All these subclasses differ in epidemiology, severity, disease mechanisms, and therapeutic response to treatments [7].

Furthermore, the thymus plays a crucial role in AChR-mediated MG. EOMG exhibits a high frequency of hyperplastic thymus and high rates of comorbidity with other autoimmune diseases. Typically, thymectomy is the best treatment option for patients who have this subtype of MG, improving clinical outcomes. In contrast, LOMG generally exhibits a normal or atrophic thymus and a worse response to thymectomy [8]. MG is the paraneoplastic disease most associated with thymoma, a neoplasm of the thymus originating from its epithelial tissue.

Thymomas are seen in 15–30% of MG cases, typically among men over the age of 40, giving rise to one of the most severe forms of the disease among patients with detectable serum AChR autoantibodies [9]. In addition, a significant percentage of thymoma patients develop MG or exhibit AChR autoantibodies without disease manifestations [8].

Intriguingly, EOMG is more common in females, exhibiting a sex ratio of 3:1 in the 30–50 age group. By contrast, the onset of the disease over the age of 50 (LOMG) is more common in males (3:2), with the highest rates between the ages of 60 and 89. The incidence rate remains approximately the same during puberty, while pediatric MG is very rare [4,7,10].

Although the factors leading to gender disparities in the onset of MG remain unclear, the involvement of sex hormones and the X chromosome in the female predominance of several autoimmune diseases has been largely corroborated [1,11,12,13,14,15,16].

Inactivation of the X chromosome (XCI) is an epigenetically regulated dosage compensation mechanism in female mammalian cells. This phenomenon occurs during early development, resulting in transcriptional silencing of the X chromosome of paternal or maternal origin in the same nucleus. Thus, females are mosaics for X-linked genes due to the two different cell populations, each with different functional parental chromosomes [17].

The choice of the X chromosome to inactivate is generally random, with a 50:50 ratio between the active X chromosomes derived from parents. However, females often have preferential or skewed XCI, in which the vast majority (>75–90%) of all cells within a tissue inactivate the same parental X chromosome. Asymmetric XCI may result either from the stochastic event during the commitment of X inactivation (Xi) or from negative selection against structural abnormalities or mutations involving an X chromosome that potentially affects cell survival. In addition, the XCI status is dynamic over the course of life: a skewed pathway can establish a non-pathogenic acquired trait in older women following stochastic clonal loss or selection [18].

It is worth considering that, since the X chromosome is enriched with immune-related genes, skewed XCI patterns could influence thymic tolerance induction processes—thus increasing the risk of developing autoimmunity [19]. Coherently, the contribution of the X chromosome in the strong female preponderance of autoimmune diseases onset has been reported [20].

For example, female patients with primary biliary cirrhosis (PBC), systemic lupus erythematosus (SLE), Sjogren’ syndrome (SS), or autoimmune thyroiditis (AITD) are seven to ten times more affected than males. These disparities weaken in rheumatoid arthritis (RA) or systemic sclerosis (SSc) pathologies (female to male ratio 2–3:1) [12,21,22].

Unbalanced dosage of X-linked genes with immunological function—such as *CXorf21*, *TLR7*, and other genes known to escape to XCI, or aberrant expression of *XIST*—contribute to the pathogenesis of SLE or SS [23,24].

Furthermore, the biased prevalence of XCI was investigated in SS and RA, revealing a strong pattern of asymmetric inactivation of XCI associated with the presence of HLA-DRB1 susceptibility alleles in RA patients [25].

The skewed pattern of XCI was also one of the proposed factors associated with AITD in both adults and early-onset forms. Patients with Hashimoto thyroiditis (HD) show a greater frequency of skewing than patients with Graves’ disease (GD) in childhood. Loss of XCI mosaicism in AITD was also found to be particularly pronounced in severe forms of both HD and GD compared to medium or remission forms. However, the data are not yet consistent to confirm the relationship between the XCI pattern and the severity or activity of the diseases [26,27,28].

Therefore, preferential or skewed XCI, as well as escape from the X inactivation of some immune-related genes, could contribute to predisposing females to immunodeficiency syndromes.

No data on MG is yet available. Therefore, we hypothesize that an enhanced skewed XCI pattern may contribute to the female bias observed in the onset of MG, especially among younger women; we performed the present study to test this hypothesis.

## 2. Materials and Methods

### 2.1. Study Population

A total of 284 women between the ages of 20 and 82 were originally recruited for the present study. Informative results for X-chromosome inactivation were obtained in 251 of them, consisting of 121 female patients with MG (mean age 47.90 ± 12.15 years; range 26–82 years) and 130 age-matched controls (mean age 45.82 ± 11.75 years; range 20–74 years) (Table 1). The remaining 33 women—18 MG patients and 15 controls—were homozygous at the HUMARA locus and were therefore excluded for not being informative for XCI. The 121 AChR positive myasthenic female patients (AChR+) were recruited at the Myasthenia Clinic, Department of Neuroscience and Cardiac and Thoracic Department of the Pisa University Hospital. All recruited patients underwent neurological and serological evaluation, and the diagnosis of MG was based on clinical symptoms together with positivity to AChR antibodies. All the patients were treated with steroids, and 64% of them were also treated with Mestinon at the time of recruitment. We also recorded immunosuppressant treatment in nine patients. The severity of MG was assessed using the Osserman classification. All patients underwent chest computed tomography (CT) followed by thymectomy. Histological results showed a normal (involuted) thymus in 20 patients, follicular hyperplasia in 18, and thymoma in 82 (Table 1).

The control population consisted of 130 age-matched volunteers with no personal history of autoimmune disease recruited at the S. Paolo Hospital in Milan. Peripheral blood samples from case and control women were collected in EDTA tubes and stored until testing. The study was conducted according to the Declaration of Helsinki, and the Ethics Committee of the Pisa University Hospital approved the protocol (Protocol number 51193/2018).

### 2.2. Assessment of X-Chromosome Inactivation (XCI)

The XCI pattern was established in the peripheral blood lymphocyte (PBL) DNA of patients and controls by analysing the methylation status of a polymorphic locus in the androgen receptor gene (HUMARA assay), as previously reported [29,30].

Briefly, PCR was performed after double enzymatic digestion with the methylation-sensitive restriction enzymes *HpaII* and *HhaI* (Boehringer Ingelheim, Ingelheim, Germany).

**Figure 1:**
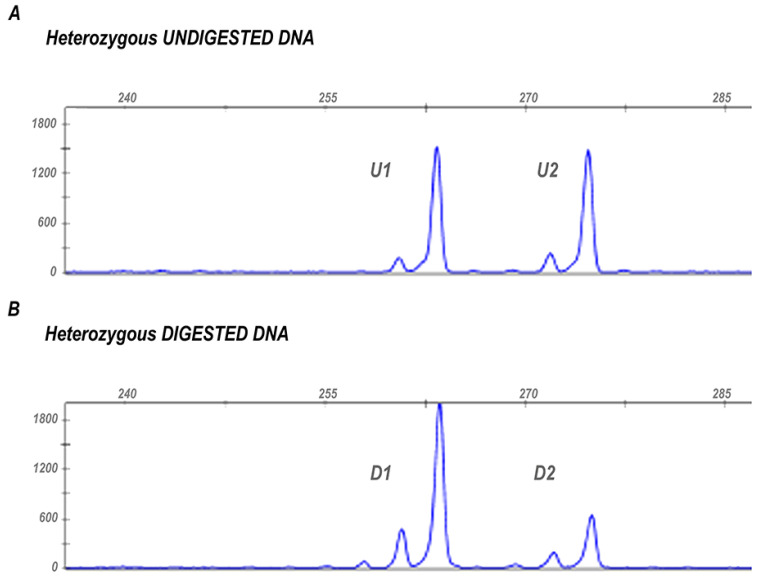
X chromosome inactivation pattern assessed through the HUMARA assay in an informative sample. (**A**) The figure shows the two peaks (U1 and U2) generated by the amplification of undigested *AR* alleles in a heterozygous subject. The different length of the two PCR products results from the polymorphic microsatellite located within the amplified region that allows the discrimination between the two parental *AR* alleles. (**B**) Peaks generated by the two *AR* alleles after digestion with *HpaII* and *HhaI* restriction enzymes (D1 and D2). The different areas of the peaks, before and after digestion, are used to estimate XCI ratio.

The XCI pattern is estimated based on *HpaII* and *HhaI* restriction sites located in exon 1 of the *AR* gene. These sites are methylated on the inactive X chromosome and un-methylated on the active one. The methylation of the restriction sites on the inactive X chromosome protects them from digestion so that only the methylated *AR* allele is not digested and subsequently amplified during the PCR reaction. Informative samples are heterozygous for a polymorphic CAG repeat located within the amplified region, allowing for distinguishing between the two parental alleles (Figure 1).

Each sample was tested in duplicate, and a male sample was included in each experiment to verify complete enzymatic digestion. The PCR products were separated by capillary electrophoresis using the ABI PRISM 3100 Genetic Analyzer (Thermofisher Scientific, MA, USA) and analysed by the GeneScan software to quantify the PCR product from each allele by peak area.

The XCI pattern is generally random, i.e., on average, 50% of cells display one of the two active *AR* alleles. However, in some cases, one allele is inactivated in most cells. The cut-off defining skewed XCI was defined as ≥75% [21,31] or ≥80% [28] and severely skewed when the XCI ratio was ≥90% [21]. We calculated X-inactivation ratios in heterozygotes using the following formula indicated by [32]:

(D1/U1)/(D1/U1 + D2/U2)

In the formula reported, D1 and D2 represent the two peak areas from the digested sample, and U1 and U2 are the corresponding areas of the alleles obtained from undigested DNA (to correct for preferential amplification of smaller alleles) (Figure 1).

Finally, we considered cut-off values of ≥75% defining moderate skewed XCI and ≥90% for severe skewed XCI, as previously reported [21].

### 2.3. Statistical Analyses

The chi-squared test and Fisher’s exact test were used to compare the XCI patterns of patients and controls. Any *p*-values < 0.05 were considered statistically significant. The Yates’ correction was applied to the obtained *p*-values.

## 3. Results

### Quantification of the Degree of XCI Skewing in MG Patients

The XCI distribution patterns in patients and controls are shown in Table 2.

Among the 121 female patients with MG, 53% (64/121) had random XCI vs. 83% (108/130) among controls.

In the general population, we detected moderate skewed inactivation in 14% (18/130) of women considered, while only 3% (4/130) of them showed severe skewed XCI. Moreover, a severe skewed XCI was peculiar of aged women (all the four women showing severe XCI were aged > 45 years).

Among myasthenic women, 21% (28/121) were characterized by moderate skewed XCI, and 24% (29/121) showed severe skewed XCI. Severe skewed inactivation was equally distributed among younger and older MG women: 11% (13/121) women aged < 45 years and 13% (16/121) aged ≥ 45 years.

Considering the low number of control women who showed an extremely unbalanced pattern of XCI, we decided not to subdivide XCI into moderate and severe. Thus, we regarded only the cut-off value of ≥75% to define skewing XCI.

Given this premise, the frequency of skewed XCI pattern in the MG population (47% (57/121)) was significantly higher (*p* = < 0.00001) than in healthy controls (22/130, 17%) (Table 2).

The final distribution of XCI patterns in patients and controls is shown in Table 3.

When comparing MG women < 45 years of age (early-onset MG; 27/62, 43.5%) with matched control women (4/69, 6%), we observed a highly significant increase of skewed XCI pattern (*p* = <0.00001) in myasthenic women (Table 3). This was not observed in women over 45 years of age at sampling, although a significant increase in skewed XCI pattern (*p* = 0.03) was still observed in MG women (30/59, 50%) compared to controls (18/61, 29.5%) (Table 3). This is because the control group of women showed a significant increase in skewed XCI patterns with increasing age at sampling (4/65, 6% in those aged < 45 years vs. 18/61, 29.5% in those over 45 years of age (*p* = 0.0007)), while a large number (27/62, 43.5%) of MG females showed a skewed XCI pattern already at an early age, not significantly different from that observed in older female MG patients (aged ≥ 45 years) (27/62, 43.5% vs. 30/59, 51%; *p* = 0.53) (Table 3).

**Figure 2:**
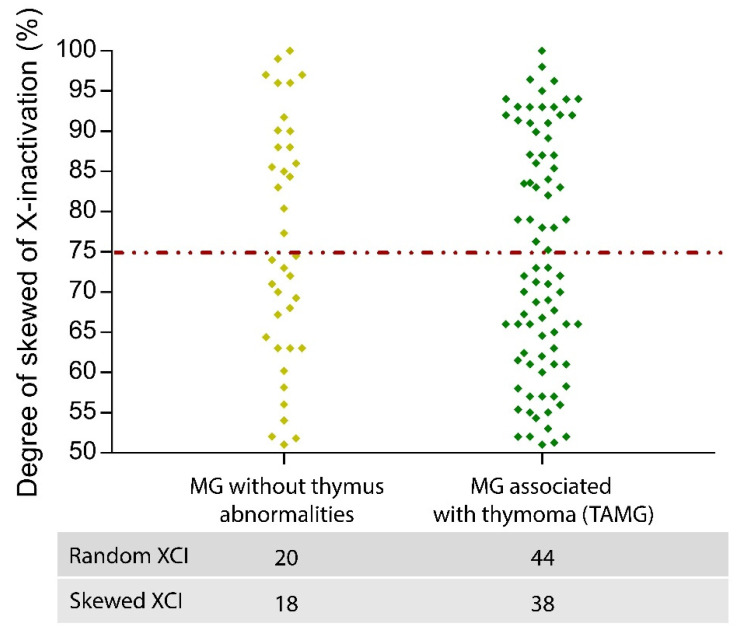
Distribution pattern of XCI in MG women with and without thymus abnormalities. The distribution of XCI patterns in the two MG subgroups is shown. Compared with 45% of TAMG, 47% of MG showed skewed inactivation. Therefore, it was not possible to detect significant differences in the chromosomal inactivation of MG and TAMG patients.

The comparison of female patients without thymoma with TAMG females showed no difference in the pattern of skewed XCI (*p* = 0.92), suggesting that skewed XCI is not a risk factor for TAMG (Figure 2). Moreover, we investigated the age-related XCI pattern of MG onset (<45 years vs. ≥45 years) exclusively in women without thymoma manifestation, but no significant differences were found (*p* = 0.90). Finally, no significant differences were found in skewed XCI based on Osserman stages and the age of onset of MG (*p* = 0.77) (Table 3). All MG females were under steroid treatment; therefore, we could not evaluate the effect of steroids on XCI patterns. Mestinon, an AChR inhibitor, was taken by 64% of the patients. No difference in skewed XCI patterns was seen between MG females taking Mestinon and those not taking this drug (*p* = 0.58).

## 4. Discussion

It is estimated that about 80% of autoimmune syndromes have a strong female bias, and SLE, SS, or, AITD have undeniable female prevalence, reaching a sex bias of 10–7:1 [33]. As an autoimmune disorder, MG exhibits female preponderance, especially in early-onset cases [7].

The lack of investigation into the involvement of the XCI in MG made it necessary to continue research and fully elucidate the mechanisms explaining the sex bias observed in the disease.

XCI is a peculiar compensation mechanism of female dosage that could be instrumental in female predisposition to autoimmune diseases. For example, some genes that can escape from XCI are mostly involved in the innate and adaptive immune response [14].

In this study, we hypothesized the contribution of a skewed XCI to the etiology of the disease and the female predisposition to MG. The preferential X mosaic condition was established in the peripheral blood of 121 MG and 130 healthy women using HUMARA assay.

As the degree of XCI skewing detected in our control group showed a bimodal distribution, we considered XCI with ≥75% skewing as “skewed XCI.”

Our results revealed that asymmetric inactivation of the X chromosome was significantly more frequent among myasthenic women than among healthy individuals (47% vs. 17% respectively; *p* = < 0.0001).

This result is consistent with the observation of increasing allelic imbalance across the entire X chromosome in SLE lymphocytes, particularly at the level of genes that escape the X inactivation with both protective and predisposing roles to disease onset [24].

A skewed pattern was also reported in 34% of women with HT and in 31% of women with GD, compared with 8% in healthy controls [34]. Comparable results were obtained in an independent study conducted by Chabchoub and collaborators (2009), considering both AITD and RA: they also reported a significant association between extremely skewed XCI and RA [35].

Thus, the contribution of skewed XCI has been extensively investigated in autoimmune thyroiditis, scleroderma, RA, juvenile idiopathic arthritis, and multiple sclerosis [25,26,27,28,36,37,38]. In this regard, our findings contribute to the evidence supporting the implication of altered XCI features in the pathogenesis of many autoimmune diseases.

Notably, younger myasthenic women showed a significant increase in the skewed XCI pattern compared to controls, the latter group being characterized by a random Xi within the first four decades of life and a shift to a skewed pattern during life. This result was further reinforced (*p* = < 0.00001) when we considered only cases and controls aged < 45 years, suggesting a strong contribution of skewed XCI in EOMG. Indeed, early manifestations of the disease occur mainly before 45 years of life, defined as EOMG, and characterized by the strongest female preponderance [39,40].

Although epidemiological studies show a higher prevalence of MG in young women, we found no difference in skewed XCI patterns when comparing the EOMG and LOMG subgroups. By contrast, in control women, there was a significant increase in skewed XCI frequency in women aged more than 45 years compared to younger women. Furthermore, a significant difference in skewed XCI was observed also comparing MG and control women aged more than 45 years at sampling, suggesting that, albeit to a lesser extent than in EOMG, a skewed XCI pattern can still contribute to the disease in aged women.

We next investigated if there was a preferential skewed XCI pattern in MG females with or without thymoma or stratified according to the Osserman classification of MG. Present results showed no difference among groups, suggesting that the skewed inactivation pattern of the X chromosome does not contribute to disease severity or to the risk of developing a thymoma in MG females.

To date, the actual prevalence of skewed XCI in unaffected populations is still debated. However, the physiological increase of preferential XCI associated with aging has been widely reported in several independent studies indicating that the acquired skewed XCI is a highly prevalent phenotype after 50–60 years of age, particularly in blood tissues [37,41,42,43,44,45,46,47].

Our results are in line with the studies mentioned above, as age-acquired skewed XCI could influence the predisposition and manifestation of aging-related traits, such as hematopoietic malignancy in women [46,48].

Furthermore, we cannot confirm the role of a skewed XCI pattern in the manifestation of thymoma. Previous works have discussed the involvement of X chromosome inactivation or X chromosome loss in predisposing to different types of cancer, as well as the protective role of EXIST (Escape from X-inactivation Tumour Suppressor) genes in female cancer onset [49,50]. For instance, a skewed pattern of XCI was observed in the blood cells of patients with aggressive oesophageal or lung cancers [51,52], while X chromosome loss and skewed XCI have been observed in most ovarian cancers [53,54,55,56].

Kristiansen and colleagues investigated the XCI in breast cancer, revealing a higher frequency of skewed XCI in women aged 27 to 45 years compared to matched controls, while the likelihood of carrying the *BRCA1* or *BRCA2* mutation is unrelated to the XCI pattern. However, an independent study has associated skewed XCI with advanced age at ovarian and breast cancer diagnosis among *BRCA1* mutation carriers [32,57,58].

On the other hand, a skewed XCI rate was observed with similar frequencies in glioma and cervical cancer patients compared with controls, although the XCI pattern was proposed as a susceptibility biomarker in young female patients with high-grade glioma [59,60].

An increasing body of evidence investigates genetic, hormonal, microbial, and environmental factors contributing to the sex-specific susceptibility to certain autoimmune conditions [1]. Oestrogens can affect anti-inflammatory and pro-inflammatory responses, depending on dose, timing, and microenvironment. For instance, thymic transcription factors—such as the autoimmune regulator (AIRE)—could be influenced by sex hormones, modulating the risk of developing MG with AChR antibodies [61].

Furthermore, the X chromosome plays a fundamental role in autoimmunity, as it contains genes involved in maintaining physiological levels of sex hormones and genes with established roles in autoimmunity.

The dynamic of the X chromosome is mediated by epigenetic mechanisms: X inactivation, X monosomy, and the escape of several genes from chromosome silencing are peculiar traits that could lead to immune deregulation [14,21,44,62].

A presumed explanation for the asymmetric involvement of XCI in autoimmunity is that the loss of mosaicism could result in the escape of X-linked self-antigens presentation in the thymus, affecting self-tolerance and consequently the development of autoimmune diseases. The unbalanced XCI process increases the tendency to generate self-reactive lymphocytes, together with a reduced ability to suppress or eliminate them [19,63].

Furthermore, transcripts of the X-chromosome regulate the transcription of autosomal genes, influencing molecular pathways such as DNA methylation, glucose, and protein metabolism with sex-specific outcomes [64].

Additionally, a recent investigation identified 15 significant miRNA clusters associated with MG, five of which were associated with immunity. Interestingly, hsa-miR-92a-3p, hsa-miR-363-3p, hsa-miR-20b-5p, and hsa-miR-18b-3p are located on the X chromosome [65].

Even though our work fits well in the context analysed, we are aware that this study has some limitation points that need further investigation. In detail, we are unable to propose a comparison between preferential inactivation of X in patients undergoing pharmacological treatment and treatment naïve patients. All the women in our cohort underwent steroids treatment, while a minor percentage took Mestinon and immunosuppressants. Although the absence of treatment-naïve patients does not allow us to evaluate the potential effects of steroids on skewed patterns detected, we observed no difference in XCI between women taking Mestinon and women not taking it.

However, the characterization of the HUMARA locus makes it a reliable target for determining the state of inactivation of the X chromosome. The use of other loci that map to the X chromosome is not recommended because of the sensitivity of methylation to various factors such as drugs, effects of mitosis, nutrition, and more [66].

Moreover, the double digestion with two methylation-sensitive restriction enzymes minimizes the slight variation in methylation pattern that could occur among the target sites [67].

In addition, several previous studies investigating the potential pharmacological effect on the HUMARA locus methylation considered it unlikely that immunosuppressant, steroid, hormonal, or psychiatric treatment induced the skewed XCI in patients [25,28,36,38,68,69]. In particular, Broen and colleagues demonstrated that treatment with steroids had no effect on the detection of XCI assumed with HUMARA assay in patients with SSc [69].

However, considering that this is the first evidence of preferential XCI in MG, further investigations are needed prior to definitively excluding any pharmacological contribution to the observed phenomenon.

The present study revealed a strong increase in skewed XCI pattern in EOMG females compared to matched controls, suggesting that it can contribute to the female preponderance of the disease reported in the EOMG subgroup. However, despite there being an age-related increase in skewed XCI among controls, we still observed a slightly significant increase of skewed XCI in aged MG females compared to aged controls. Further studies are therefore required to clarify the molecular consequences of a skewed XCI in females with MG in terms of altered gene expression levels and their contribution to the disease.

## 5. Conclusions

Preferential inactivation of a parent-derived X chromosome is related to female preponderance of several autoimmune diseases. Our work first proposes the involvement of XCI dynamics in the etiology of MG, particularly among young women.

## Figures and Tables

**Table 1:** Characteristics of cases and controls.

**Patients**				
**No of Patients**	**Age** **(Mean ± SD)**	**Age of Onset**	**Osserman** **Classification**	**Thymic Histology**
Informative *n* = 121	47.90 ± 12.15	<45 years *n* = 74	I *n* = 4	No thymoma *n* = 20
		≥45 years *n* = 45	II *n* = 96	Hyperplasia *n* = 18
		n.a. *n* = 2	III *n* = 13	Thymoma *n* = 82
			IV *n* = 6	n.a. *n* = 1
			n.a. *n* = 2	
**Controls**				
**No of Controls**	**Age** **(Mean ± SD)**			
Informative *n* = 130	45.82 ± 11.75			

n.a. = not available data.

**Table 2:** Distribution of XCI pattern in case and control population.

	Random XCI < 75%	Moderate XCI ≥ 75%	Severe XCI ≥ 90%
MG patients	64	28	29
Control population	108	18	4

**Table 3:** Distribution of XCI pattern in patients and controls (“MG/Control aged” is referred to age at blood collection). The chi-squared test and Fisher’s exact test were used, and the Yates’s correction was applied to the *p*-values obtained. Statistically significant results are marked with (*).

	Random XCI (XCI < 75%)	Skewed XCI (XCI ≥ 75%)	*p*-Value
MG patients	64	57	<0.00001 (*)
Controls	108	22	
MG aged < 45 years	35	27	0.53
MG aged ≥ 45 years	29	30	
Controls aged < 45 years	65	4	0.0007 (*)
Controls aged ≥ 45 years	43	18	
MG aged < 45 years	35	27	<0.00001 (*)
Controls aged < 45 years	65	4	
MG aged ≥ 45 years	29	30	0.03 (*)
Controls aged ≥ 45 years	43	18	
Age of onset of MG			
<45 years	42	32	0.77
≥45 years	21	24	
Osserman stages			
I	3	1	0.63
II	51	45	
III	7	6	
IV	2	4	

## Data Availability

The datasets generated and/or analyzed during the current study are available from the corresponding author on reasonable request.
